# Treatment of one case of cerebral palsy combined with posterior visual pathway injury using autologous bone marrow mesenchymal stem cells

**DOI:** 10.1186/1479-5876-10-100

**Published:** 2012-05-18

**Authors:** Min Li, Aixue Yu, Fangfang Zhang, GuangHui Dai, Hongbin Cheng, Xiaodong Wang, Yihua An

**Affiliations:** 1 Department of stem cell transplantation, General Hospital of Chinese People’s Armed Police Forces, Beijing, 100039, China; 2 Beijing Neurosurgical Institute, Beijing Tiantan Hospital, Capital Medical University, Beijing, 100050, China

## Abstract

**Background:**

Cerebral palsy is currently one of the major diseases that cause severe paralysis of the nervous system in children; approximately 9–30% of cerebral palsy patients are also visually impaired, for which no effective treatment is available. Bone marrow mesenchymal stem cells (BMSCs) have very strong self-renewal, proliferation, and pluripotent differentiation potentials. Therefore, autologous BMSC transplantation has become a novel method for treating cerebral palsy.

**Methods:**

An 11-year-old boy had a clear history of dystocia and asphyxia after birth; at the age of 6 months, the family members observed that his gaze roamed and noted that he displayed a lack of attention. A brain MRI examination at the age of 7 years showed that the child had cerebral palsy with visual impairment (i.e., posterior visual pathway injury). The patient was hospitalized for 20 days and was given four infusions of intravenous autologous BMSCs. Before transplantation and 1, 6, and 12 months after transplantation, a visual evoked potential test, an electrocardiogram, routine blood tests, and liver and kidney function tests were performed.

**Results:**

The patient did not have any adverse reactions during hospitalization or postoperative follow-up. After discharge, the patient could walk more smoothly than he could before transplantation; furthermore, his vision significantly improved 6 months after transplantation, which was also supported by the electrophysiological examinations.

**Conclusions:**

The clinical application of BMSCs is effective for improving vision in a patient with cerebral palsy combined with visual impairment.

## Background

This case study represents a typical case of cerebral palsy combined with visual impairment. With medical advances, the active treatment of preterm infants, children with dystocia, and children with encephalitis or hypoxic-ischemic encephalopathy results in significant increases in the survival rates of these patients; however, compared to normal infants, these patients have significantly increased incidences of periventricular white matter demyelination. Many regions of the brain play important roles in the generation of vision, such as the cerebral cortex, the white matter, and the basal ganglia [1-3]. During the perinatal period, most cerebral palsy patients have a history of intrauterine hypoxia, preterm birth, dystocia, asphyxia after birth, kernicterus, or other complications. These factors can all result in impaired function of the visual areas of the brain, which currently has no effective treatment.

## Methods

### Ethics

Prior to stem cell therapy, a consent form was signed by the guardians of the patient. This study was approved by the ethics committee of our hospital.

### General patient information

#### General information

The patient, an 11-year-old boy, was admitted to our hospital mainly due to “binocular vision disorders that had lasted more than 11 years”. The patient was born full-term with dystocia and had a history of significant asphyxia, which improved after symptomatic treatment. At the age of 6 months, his family members found that he had trouble concentrating and following objects as well as easy irritability and hyperactivity. A brain MRI performed at the age of 7 showed cerebral hypoplasia and optic nerve atrophy. The diagnosis was cerebral palsy with visual impairment. He could not see and had no light perception before he was 8 years old. At the age of 8, the patient started to have light perception, and he could only see objects 20 cm in front of him at the age of 9; after that, his vision did not improve. When admitted to our hospital, the physical examination revealed that the patient presented with stuttering, a right-eye visual acuity of 30 cm, and a left-eye visual acuity of 20 cm. The pupils were equal and round with diameters of 3 mm; the eyes were sensitive to light reflection, the movements of both eyes were normal, strabismus was absent, horizontal nystagmus was present, and eye closure was effective. The bilateral finger muscle strength was grade 4, the right lower limb muscle tension was grade 1, the tendon reflexes were active, and the bilateral Chaddock’s sign was (+).

#### Auxiliary examination

**Figure 1:**
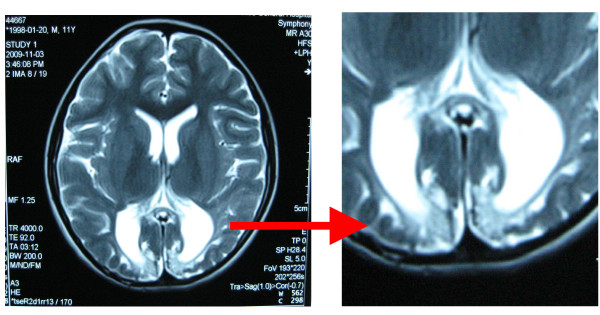
**Brain MRI showed the bilateral parietal-occipital lobe and corpus callosum dysplasia**.

A brain MRI examination revealed bilateral parietal-occipital lobe and corpus callosum dysplasia (Figure 1).

Ocular fundus examination: A leopard-spot-like fundus was observed, the boundary of the optic disc was clear, the color of the temporal disc border was light, the C/D was 0.5–0.6 (normal cup to disc ratio: <0.3), and the fovea reflex was (−).

#### Visual evoked potential (VEP) (2010-10-22)

**Figure 2:**
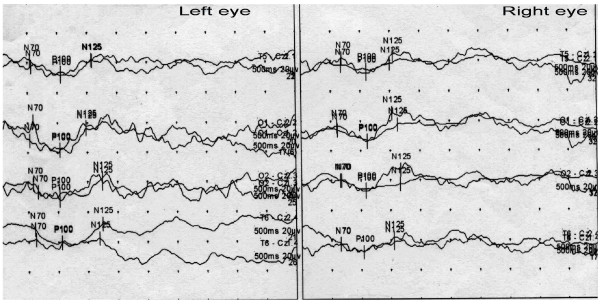
**Visual evoked potential on Oct. 22, 2010: Flash stimulation tests were performed on both eyes.** The results revealed that the waveforms of both eyes were poorly differentiated, the fundus was slightly wider than normal; P100 amplitude was lower than normal (T6- CZ), but the P100 latency was basically normal.

Flash stimulation tests were performed on both eyes. The results revealed that the waveforms of both eyes were poorly differentiated, the fundus was slightly wider than normal, and the P100 latency was basically normal (Figure 2).

### Intervention measures

#### Stem cell source

Bone marrow was collected from the patient. All tests were normal, including blood leukocytes; lymphocytes; liver function (aspartate aminotransferase, alanine aminotransferase, alkaline phosphatase, lactate dehydrogenase, and hydroxybutyrate dehydrogenase); renal function (uric acid, creatinine, β2-microglobulin, and α1 microglobulin); blood sugar; and blood lipids. The patient did not have syphilis, HIV, hepatitis B, or hepatitis C infection, and there was no history of familial or hereditary diseases.

#### BMSC culture

Bone marrow was collected from the posterior superior iliac spine. Sixty microliters of bone marrow was extracted, and mononuclear cells in the bone marrow were separated using density gradient centrifugation. Cells were then inoculated into 100 ml flasks at a concentration of 3 × 10^5^ cells/ml in α-MEM containing 10% FBS. Cells were passaged when the cell density was above 90%. Cells were collected at the 5th generation and were used for transplantation by intravenous infusion.

#### The BMSC quality standard

Prior to clinical application, multiple tests were performed on the stem cells, including (1) cell morphology, (2) cell surface marker detection, (3) bacterial detection, (4) mycoplasma detection, and (5) endotoxin detection.

#### Clinical session of treatment

At each treatment session, the stem cells were fully dispersed and mixed using 100 ml normal saline, and then 4 × 10^7^ cells were intravenously infused. Four infusions were performed in each session, and the interval between two infusions was 7 days. After each time infusion, the patient was monitored for vital signs including blood pressure, heart rate, blood oxygen, and respiration over 24 hours. Immunosuppressants were not administered throughout the treatment period.

#### Monitoring of the clinical response in the patient

Blood leukocytes, lymphocytes, liver function, and kidney function were monitored before transplantation and 1, 3, 6, and 12 months following.

## Results

### Detection of BMSC quality

**Figure 3:**
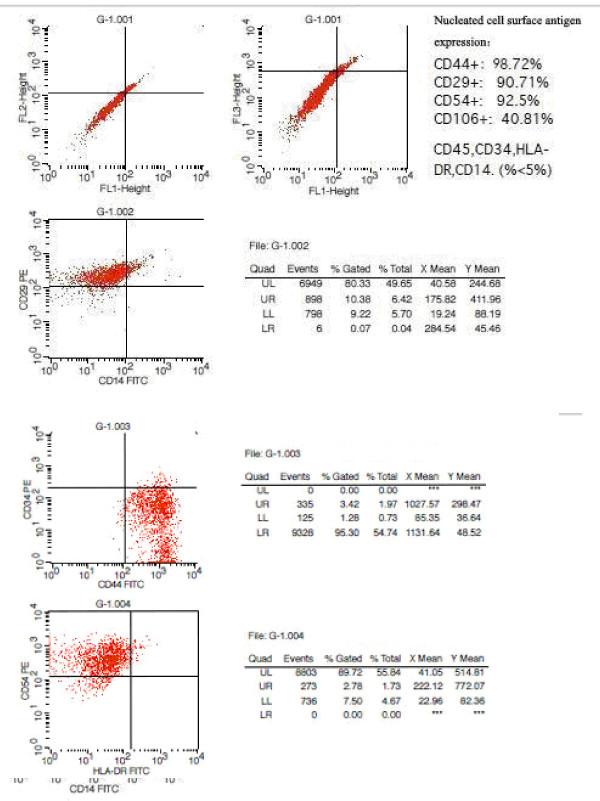
**BMSC surface marker detection: CD44 and CD29 positive rates were greater than 95%, the CD106 positive rate was greater than 80%, and the CD34, CD45, CD14, and HLA-DR expression rates were less than 5%**.

(1) Cell morphology—The cells showed typical BMSC morphology and strong growth under a phase-contrast microscope. (2) Cell surface marker detection: CD44 and CD29 positive rates were greater than 95%; the CD106 positive rate was greater than 80%; and the CD34, CD45, CD14, and HLA-DR expression rates were less than 5% (Figure 3). (3) Bacterial detection was negative. (4) Mycoplasma was negative. (5) Endotoxin results were negative as determined using limulus reagents (according to the bacterial endotoxin and bacterial endotoxin application guidelines of Chinese Pharmacopoeia, 2000 edition).

### Monitoring of adverse reactions after transplantation

**Figure 4:**
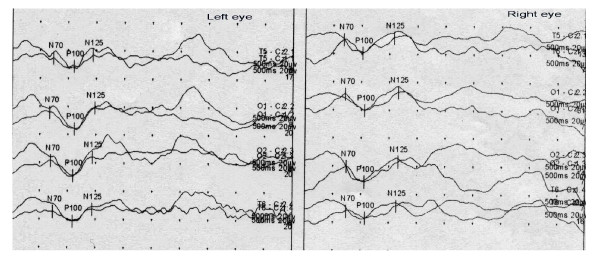
**Visual evoked potential on Oct. 12, 2011: Flash stimulation tests were performed on both eyes.** The VEP results showed that the waveforms of both eyes were well differentiated, the fundus was slightly wider, P100 amplitude increased obviously and the P100 latency was essentially normal.

After one session of stem cell transplantation and at 1, 3, 6, and 12 months after transplantation, the patient did not have any side effects or complications; all test results were within normal limits, and all vital signs were stable. A physical examination 1 year after transplantation (2011-10-12) revealed that the patient had a right-eye visual acuity of 1 m and a left-eye visual acuity of 80 cm. The right lower limb muscle tension was significantly decreased compared to that before transplantation, and the patient had heel contact with the ground and walked more smoothly than before transplantation. The auxiliary examinations were performed 1 year after transplantation (2011-10-12); the VEP results showed that the waveforms of both eyes were well differentiated, the fundus was slightly wider, and the P100 latency was essentially normal when flash stimulation tests were performed on both eyes (Figure 4).

## Conclusion

The classic visual pathway contains two parts. The first part is the anterior visual pathway, which refers to the visual pathways below the lateral geniculate nucleus, including receptors, bipolar cells, ganglion cells, the optic nerve, the optic chiasm, the optic tract, and the lateral geniculate body. The second part is the posterior visual pathway, which refers to the visual pathway posterior to the lateral geniculate body, including the optic radiation, the subcortical area, and the visual cortex. The patient in this study had a posterior visual pathway injury, which was based on (1) normal pupillary light reflex; (2) fundus examination, which showed no obvious optic atrophy; (3) eye examination, which showed no positive signs; (4) VEP after flash stimulation of both eyes, which showed poor bilateral waveform differentiation and a wider fundus; and (5) a brain MRI, which showed hypoplasia of the bilateral parietal-occipital lobe and the corpus callosum. Posterior visual pathway injuries are commonly observed in cerebral contusions of the occipital lobe and in diffused brain injuries, such as brain edemas and hernias; these injuries are usually accompanied by signs of nervous system damage. In addition to visual injuries, this patient had limb movement disorders.

BMSCs have very strong self-renewal, proliferation, and pluripotent differentiation capabilities. Under different induction conditions, BMSCs can differentiate into many types of cells *in vitro,* such as bone cells, cartilage cells, fat cells, tendons, and muscle cells. BMSCs have many advantages; they are easy to obtain and amplify *in vitro*, are unlikely to cause immune rejection after transplantation *in vivo*, and present no ethical controversy. Recent studies have also indicated that BMSCs can be induced into neuron-like cells and glial cells both *in vivo* and *in vitro*[4-6] and can differentiate into visual receptors and glial cells in the ganglion [7,8]. Many studies have evaluated the treatment of cerebral infarctions [9] and traumatic brain injuries using BMSCs; however, studies on hypoxic-ischemic encephalopathy have just begun. In some studies, BMSCs were intravenously transplanted into newborn rats with hypoxic-ischemic encephalopathy; the characteristics of transplanted stem cells could be detected in rat brains, indicating that stem cells can pass through the blood–brain barrier. In addition, these cells can survive at the sites of injury. The specific mechanism may involve several factors. First, the process of stem cells passing through the blood–brain barrier may be a specific transfer process mediated by endothelial cell adhesion molecules in the blood–brain barrier [10]. Second, after brain tissue injury, the blood–brain barrier is damaged, thus increasing permeability [11]. Third, injured tissues possess chemotaxis functions [12]. Finally, cerebral ischemia can induce the expression of neurotrophic factors around the ischemic zone, which is favorable for cell survival [13].

In this study, the characteristics of surface antigens on cultured stem cells were detected using flow cytometry. The results showed that the expression levels of CD44, CD29, CD54, and CD106 were high in nuclear cells (with expression rates of 98.72%, 90.71%, 92.5%, and 40.81%, respectively). In contrast, the cells were negative for CD45, CD34, HLA-DR, and CD14 expression (<5% expression). In BMSC studies, CD9, CD29, CD44, CD71, CD90, and CD106 are recognized as important surface markers of BMSCs, while the hematopoietic stem cell marker antigen CD3, the leukocyte marker antigen CD45, and the monocyte/macrophage surface antigen CD14 are not expressed [14,15]. CD45 is an important surface marker of MSCs [16]; it can mediate cell-cell and cell-extracellular matrix interactions and participate in the growth, maturation, and differentiation of MSCs. CD44 regulates the differentiation, fusion, and migration-specific adhesion of MSCs, thus providing anchorage-dependent growth sites for CD44-expressing MSCs. The cultured stem cells in this study were MSCs.

The mechanism underlying the function of stem cell transplantation in nerve repair is still not very clear. There are two general hypotheses: cell replacement function and neurotrophic effect. Therefore, many researchers are trying different methods to induce the differentiation of BMSCs into essential neuron-like cells for transplantation; they believe that this strategy could enable the transplanted stem cells to fully replace the damaged cells; however, from the viewpoint of neurotrophic hypothesis, pure neuron-like cells might not maximally perform the required function. We believe that the transplanted cells should not be too pure; they should consist of a mixture of a few cell types. Some cells could perform nerve repair through cell replacement, while other cells could secrete different cytokines to assist in nerve repair and regeneration, thus supporting the growth of neural stem cells and inducing differentiation [17]. Therefore, we used BMSCs without inducing any specific differentiation and performed the transplantation using intravenous fusion, which is a safe and simple method.

One year after receiving one session of BMSC transplantation, the patient described in this study showed significant improvement based on his symptoms, physical examinations, and electrophysiological studies. The physical examination results revealed that the right-eye visual acuity was 100 cm and the left-eye visual acuity was 80 cm. The right lower limb muscle tension was significantly decreased compared to the pre-surgical measurement, and the patient walked more smoothly than previously; the VEP was close to normal. During the treatment and at regular follow-ups at 3, 6, and 12 months post-transplant, the patient did not exhibit fever, headache, blood pressure fluctuations, arrhythmias, or other symptoms of discomfort without an obvious reason. During the period of stem cell transplantation, the patient did not use any neurotrophic drugs or drugs that improved microcirculation; he also did not receive hyperbaric oxygen therapy or participate in functional rehabilitation exercises. Of course, we cannot completely rule out the possibility that the patient self-recovered; however, it is difficult to attribute these improvements to self-recovery, as his vision did not improve significantly in the two years prior to transplantation but improved rapidly within 1 year after stem cell transplantation. Therefore, we have reason to believe that it was stem cell therapy that promoted the recovery of his vision.

The mechanism that BMSCs use to repair the nerve damage may involve the nutritional effect of the neurotrophic factors (such as BDNF and NGF) released by stem cells [18]. Research has shown that BMSCs can improve neurotrophic factor expression, which may be the key to promoting brain function recovery. Rat BMSCs can continuously express NGF and BDNF when they are cultured in vitro to the sixth generation [19,20]. In addition, BMSCs can secrete a large amount of useful cell factors, such as BDNF, that can promote B lymphocytes to increase the free calcium ion concentrations in cells and thus improve their immune regulation and resistance [21]. Mahmood [22] used BMSCs to treat rats with brain injury and showed that the concentrations of NGF and BDNF in the treatment group were significantly higher than those in the control group; Additionally, these rats still showed increased BDNF expression 3 months later. Furthermore, the mechanism may also involve the induction of angiogenesis due to the release of vascular endothelial growth factors that is promoted by stem cells, thus improving blood circulation in the injured regions [23], or a microenvironment that is favorable for the regeneration and repair of autologous stem cells provided by exogenous stem cells, thus promoting the proliferation and differentiation of autologous stem cells and repairing injured nerves. Of course, these are only hypotheses; further confirmation through basic experimental studies is needed.

The reason for the improvement in our patient’s symptoms 1 year after stem cell transplantation is unclear; it is possible that in addition to the short-lasting effects that occurred via the trophic support of MSCs, these cells exhibited long-lasting effects [24,25]. One possible explanation for the therapeutic mechanism of MSCs is that they increase neurogenesis. Preclinical studies have shown the importance of neurogenesis in an animal model of stroke, and transplanted MSCs might enhance this process [26-28]. Stroke-induced neurogenesis was reported to continue for up to 1 year and to occur in aged brains [29]. After several preclinical studies, Zhu et al. [30] treated several patients by applying autologous stem cells. These authors labeled neural stem cells from humans with superparamagnetic iron oxide nanoparticles and tracked these cells using magnetic resonance imaging (MRI). They did not observe a hypointense signal after 7 weeks, and they attributed this result to a dilution of the signal due to cell proliferation. For several reasons, including ethical concerns, it is difficult to observe stem cell survival in the human body. Currently, the only way to judge cell survival is by an improvement in the symptoms of the treated patients.

In summary, BMSC transplantation can be used to effectively treat cerebral palsy combined with visual impairment. To a certain extent, it can improve limb movement disorders and visual impairment caused by posterior visual pathway injury.

## Competing interests

The authors declare that they have no competing interests.

## Authors’ contributions

ML carried out the design of the study and drafted the manuscript. AY and FZ carried out the data collection. GD and HC participated in the design of the study. XW participated in the stem cell clinical register. YA conceived of the study, participated in its design and coordination, and helped to draft the manuscript. All authors read and approved the final manuscript.

## References

[B1] Grönqvist S Flodmark O Tornqvist K Edlund G Hellström A Association between visual impairment and functional and morphological cerebral abnormalities in full-term children Acta Ophthalmol 2001 79 140 146 10.1034/j.1600-0420.2001.079002140.x 11284751

[B2] Dutton GN Jacobson LK Cerebral visual impairment in children Sem Neonatol 2001 6 477 485 10.1053/siny.2001.0078 12014888

[B3] Mercuri E Atkinson J Braddick O Anker S Cowan F Rutherford M Pennock J Dubowitz L Basal ganglia damage and impaired visual function in the newborn infant Arch Dis Child Fetal Neonatal Ed 1997 77 F111 F114 10.1136/fn.77.2.F111 9377131 PMC1720693

[B4] Woodbury D Schwarz EJ Prockop DJ Adult rat and human bone marrow stromal cells differentiate into neurons J Neurosci Res 2000 61 364 370 10.1002/1097-4547(20000815)61:4<364::AID-JNR2>3.0.CO;2-C 10931522

[B5] Woodbury D Schwarz EJ Prockop DJ Black IB Neurospheres induced from bone marrow stromal cells are multipotent for differentiation into neuron, astrocyte, and oligodendrocyte phenotypes Biochem Biophys Res Commun 2004 322 918 922 10.1016/j.bbrc.2004.07.201 15336551

[B6] Mori T Kiyono T Imabayashi H Takeda Y Tsuchiya K Miyoshi S Makino H Matsumoto K Saito H Ogawa S Sakamoto M Hata J Umezawa A Combination of hTERT and bmi-1, E6, or E7 induces prolongation of the life span of bone marrow stromal cells from an elderly donor without affecting their neurogenic potential Mol Cell Biol 2005 25 5183 5195 10.1128/MCB.25.12.5183-5195.2005 15923633 PMC1140572

[B7] Tomita M Adachi Y Yamada H Takahashi K Kiuchi K Oyaizu H Ikebukuro K Kaneda H Matsumura M Ikehara S Bone marrow-derived stem cells can differentiate into retinal cells in injured rat retina Stem Cells 2002 20 279 283 10.1634/stemcells.20-4-279 12110696

[B8] Kicic A Shen WY Wilson AS Constable IJ Robertson T Rakoczy PE Differentiation of marrow stromal cells into photoreceptors in the rat eye J Neurosci 2003 23 7742 7749 12944502 10.1523/JNEUROSCI.23-21-07742.2003 PMC6740611

[B9] Li Y Chopp M Chen J Wang L Gautam SC Xu YX Zhang Z Intrastriatal transplantation of bone marrow nonhematopoietic cells improves functional recovery after stroke in adult mice J Cereb Blood Flow Metab 2000 20 1311 1319 10.1097/00004647-200009000-00006 10994853

[B10] Guan XQ Yu JL Li LQ Liu GX Study on mesenchymal stem cells entering the brain through the blood–brian barrier Zhonghua Er Ke Za Zhi 2004 42 920 923 15733363

[B11] Wang L Li Y Chen X Chen J Gautam SC Xu Y Chopp M MCP-1, MIP, IL-8 and ischemic cerebral tissure enhance the human bone marrow stromal cell migration in interface culture Hematology 2002 7 113 117 10.1080/10245330290028588 12186702

[B12] Dormady SP Bashayan O Dougherty R Zhang XM Basch RS Immortalized multipotential mesenchymal cells and the hematopoietic microenvironment J Hematother Stem Cell Res 2001 10 125 140 10.1089/152581601750098372 11276366

[B13] Abe K Therapeutic potential of neurotrophic factors and neural stem cells against ischemic brain injury J Cereb Blood Flow Metab 2000 20 1393 1408 11043902 10.1097/00004647-200010000-00001

[B14] Mahmood A Lu D Wang L Chopp M Intracerebral transplantation of marrow stromal cells cultured with neurotrophic factors promotes functional recovery in adult rats subjected to traumatic brain injury J Jeurotrauma 2002 19 1609 1617 10.1089/089771502762300265 12542861

[B15] Im GI Kim DY Shin JH Hyun CW Cho WH Repair of cartilage defect in the rabbit with cultured mesenchymal stem cells from bone marrow J Bone Joint Surg Br 2001 83 289 294 10.1302/0301-620X.83B2.10495 11284583

[B16] Terada N Hamazaki T Oka M Hoki M Mastalerz DM Nakano Y Meyer EM Morel L Petersen BE Scott EW Bone marrow cells adopt the phenotype of other cells by spontaneous cell fusion Nature 2002 416 542 545 10.1038/nature730 11932747

[B17] An YH Wan H Zhang ZS Wang HY Gao ZX Sun MZ Wang ZC Effect of rat Schwann cell secretion on proliferation and differentiation of human neural stem cells Biomed Environ Sci 2003 16 90 94 12747012

[B18] Li Y Chen J Chen XG Wang L Gautam SC Xu YX Katakowski M Zhang LJ Lu M Janakiraman N Chopp M Human marrow stromal cell therapy for stroke in rat: neurotrophins and functional recovery Neurology 2002 59 514 523 10.1212/WNL.59.4.514 12196642

[B19] Chen X Li Y Wang L Katakowski M Zhang L Chen J Xu Y Gautam SC Chopp M Ischemic rat brain extracts induce human marrow stromal cell growth factor production Neuropathology 2002 22 275 279 10.1046/j.1440-1789.2002.00450.x 12564767

[B20] Ye M Chen S Wang X Qi C Lu G Liang L Xu J Glial cell line-derived neurotrophic factor in bone marrow stromal cells of rat Neuroreport 2005 16 581 584 10.1097/00001756-200504250-00013 15812312

[B21] Zhang J Li Y Chen J Cui Y Lu M Elias SB Mitchell JB Hammill L Vanguri P Chopp M Human bone marrow stromal cell treatment improves neurological functional recovery in EAE mice Exp Neural 2005 195 16 26 10.1016/j.expneurol.2005.03.018 15904921

[B22] Mahmood A Lu D Qu C Goussev A Chopp M Long-term recovery after bone marrow stramal cell treatment of traumatic brain injury in rats J Neurosurg 2006 104 272 277 10.3171/jns.2006.104.2.272 16509501

[B23] Chen J Zhang ZG Li Y Wang L Xu YX Gautam SC Lu M Zhu Z Chopp M Intravenous administration of human bone marrow stromal cells induces angiogenesis in the ischemic boundary zone after stroke in rats Circ Res 2003 92 692 699 10.1161/01.RES.0000063425.51108.8D 12609969

[B24] Shen LH Li Y Chen J Cui Y Zhang C Kapke A Lu M Savant-Bhonsale S Chopp M One-year follow-up after bone marrow stromal cell treatment in middle-aged female rats with stroke Stroke 2007 38 2150 2156 10.1161/STROKEAHA.106.481218 17525391

[B25] Liu Z Li Y Zhang X Savant-Bhonsale S Chopp M Contralesional axonal remodeling of the corticospinal system in adult rats after stroke and bone marrow stromal cell treatment Stroke 2008 39 2571 2577 10.1161/STROKEAHA.107.511659 18617661 PMC2593106

[B26] Li WY Choi YJ Lee PH Huh K Kang YM Kim HS Ahn YH Lee G Bang OY Mesenchymal stem cells for ischemic stroke: Changes in effects after ex vivo culturing Cell Transplant 2008 17 1045 1059 10.3727/096368908786991551 19177841

[B27] Abrahams JM Gokhan S Flamm ES Mehler MF De novo neurogenesis and acute stroke: Are exogenous stem cells really necessary? Neurosurgery 2004 54 150 156 10.1227/01.NEU.0000097515.27930.5E 14683552

[B28] Lindvall O Kokaia Z Martinez-Serrano A Stem cell therapy for human neurodegenerative disorders-how to make it work Nat Med 2004 10 Suppl 42 50 10.1038/nm1064 15272269

[B29] Kokaia Z Thored P Arvidsson A Lindvall O Regulation of stroke-induced neurogenesis in adult brain–recent scientific progress Cereb Cortex 2006 16 Suppl 1 1162 1167 10.1093/cercor/bhj174 16766702

[B30] Zhu J Zhou L XingWu F Tracking neural stem cells in patients with brain trauma N Engl J Med 2006 355 2376 2378 10.1056/NEJMc055304 17135597

